# Supplementary material for: Near-Infrared 810 nm Light Affects Porifera Chondrosia reniformis (Nardo, 1847) Regeneration: Molecular Implications and Evolutionary Considerations of Photobiomodulation–Animal Cell Interaction
Source: Int J Mol Sci. 2022 Dec 23;24(1):226. doi: 10.3390/ijms24010226 (PMC9820676; doi:10.3390/ijms24010226)
Supplement: Supplementary file 1 [file ijms-24-00226-s001.zip › ijms-2005665-supplementary.pdf]

Table S1. PCR primer used for PCR reactions

Gene	Sequence	Position	Accession number
GAPDH (forward)	*5′-AAGCCACCATCAAGAAGG-3′*	882–899	KM217385.1
GAPDH (reverse)	*5′- CCACCAGTTTCACAAAGC-3*	1023–1040	KM217385.1
HSP60 (forward)	*5′-ACTATCATCTCGCCAATCG-3′*	74-92	ON814140*
HSP60 (reverse)	*5′-TTATCCACTCCACTCAACATT-3′*	173-193	ON814140*
HSP70 (forward)	*5′-TGTCAGTTCAGCAGTAGTG-3′*	536-555	ON814141*
HSP70 (forward)	*5′-TGTCAGTTCAGCAGTAGTG-3′*	636-657	ON814141*
HSP90 (forward)	*5′-ACCGCATTCATCGCTTGG-3′*	298-316	ON814142*
HSP90 (reverse)	*5′-GCTTCATCGTCACCTTCTTCC-3′*	385-406	ON814142*
NOS (forward)	*5′-CCACACTCTCCATAGCATTCT-3′*	378-392	ON814144*
NOS (reverse)	*5′-CCGTCCTCSGCGTAACAC-3′*	487-505	ON814144*
TNF (forward)	*5′-AGAAATCGCCAGAAGCAAGTTG-3′*	210–230	KR072662
TNF (reverse)	*5′-GATGAGCACATTGATAGCAGACC-3′*	264–286	KR072662
Bcl-2 (forward)	*5′-ATATTCACCAAGCCAATGC-3′*	366–384	MF380423
Bcl-2 (reverse)	*5′-CCAGACACCACTCTATCG-3′*	525–542	MF380423
Msi-1 (forward)	*5′- ATTGGAATGCTTAGTGAAATGC-3′*	428-449	MH687926
Msi-1 (reverse)	*5′- GTGTCTACCTTGCCTATTGC-3′*	598-617	MH687926
Wnt (forward)	*5′-AGTGTGATGCTGTTAGTGA-3′*	793-822	ON814145*
Wnt (reverse)	*5′-TGCGACATTGACCTTCTATA-3′*	888-908	ON814145*
Fibrillar collagen	*5′-GTCATCCAGGTCGTCAAG-3′*	1306–1323	KR072664
Fibrillar collagen	*5′-TAGGTCAATCTCTAATTCAGC-3′*	1482–1502	KR072664
FGF (forward)	*5′-CCTTCGTATCTTAGAGAATGGA-3′*	229-251	ON814143*
FGF (reverse)	*5′-ATGTTGCGGAACTTGAGA-3′*	324-342	ON814143*
Tgf3 (forward)	*5′-TGCCAATACCGTCCAAGTC-3′*	320-338	MH687919
Tgf3 (reverse)	*5′-TGCCTTCCTCCTCCTCTG-3′*	493-510	MH687919
Tgf4 (forward)	*5′-CATGAATGAAGCAATATCAACTG-3′*	6-28	MH687920
Tgf4 (reverse)	*5′-ATTTATTGTTCGTGGTTTCTTTAC-3′*	166-189	MH687920
Tgf5 (reverse)	*5′-ATTTATTGTTCGTGGTTTCTTTAC-3′*	134-153	MH687921
Tgf5 (reverse)	*5′-AGTCCTTTCGTCTTTCCTTATCG-3′*	189-211	MH687921
Tgf6 (forward)	*5′-CCACCTCTGAACAACAACAATAAC-3′*	483-506	MH687922
Tgf6 (reverse)	*5′-CTATGAGCACGGCAGCAAAG-3′*	593-612	MH687922

* In case the publication data of the present article is previous to the genebank sequence release, the FASTA sequences are reported below.

```
>Hsp60
GGACGCACGTTTTAGCTGTTAACTGGTTTaTCGCCGACTGTAAAGCAATGCAAGCTCTGA
GACATTTTTATCGACTATCATCTCGCCAATCGATGCGGAGTGCTCCTAGAATGGTGACTC
CTTGGCGATACTACGCTAAAGACATCAAGTATGGTGCAGAGGGTAGAGCTGAAATGTTGA
GTGGAGTGGATAAACTGGCTGATGCTGTTGCAGTAACTTTGGGCCCCAAGGTACAGAGTG
TGGTAACACAGAGcAGGATACACACATATGACATAGTAGTATGCTCGTCGAAAGGGGAAA
aCCGaGCAAGGAAGCGTCCATCCATCCATCAATAGCCTAATtAGCcGGACCAATTTGCCT
ATATGGCCAACTAATAGGAGAGCCCAGATAaCATTATtACATAgAATAATAGAGGTGGGA
GGATGAGATtAgTATAAAAGTCCATGAaGA
```

```
>HSP70
AATATAGAACTCAGTaAAAGATGGCGGaAAGAACAAAGGATGGTACTATTTATCaCGATAGTAATCCATCTCCTGAAGGC
CAACTAATCGGATCTGATGATACTGACCCAATACCAAAGCAATCAGACTATGCGaTAATAGGAATAGATCTGGGTACCAC
tTATTCGTGTGTTGCtATTTGGGAGAACGATCGTCCTACAGTGATTGCaAATAACATGGGCAATCGAACCACGCCTTCTT
GGGTCAGTTTTCACGAgGGGAACGTATTTGTTGGAGAAGCGGCGCTCAATAAGGGACTAAGGTTCCCAAAGAATACGGTT
TATGATGCCAAAAGAAtgaTAGGTAGACGCCTTGATGAACCTGCAATTCAAGATCGCTTGAAGAATTGGCCCTTcGCGGT
```

TGTCCAACATAGGAATAACTGCGCCTTTGAATTGGAATGTAACCATAACCAGAAAGATATTCTGCTTCCGGAGGAAATAT
CAGCTTATGTGTTAGTCAAGATGAAGCACACTGCAGAGGACTATCTTGGtCATTCTGTCAGTTCAGCAGTagTgACGGTg
CCAGCTTACtTCAACGACTCACAGAGGCAGGCaACCATTGACGCATGCTATGTTGCCGGGCTTAAGGTGGAACGTATTAT
CaACGAGCCAaCAGCTGCTGCCCTTGCTTACGGTTTAGAGAGAAATGCCGGGGCCATgGGAGCCaAAACACTACTGGtGT
ATGACTTGGGAGGAGGTACACTGGATGTCACTGTGATGACTGTGGATCGTGGAGtGTTTGAGGTGCTAAGTACATGTGGT
GACACTCAGCTTGGTGGTCAAGACTTTGATGCCAACTTAGTGGATCACTTCACCGAGGAGATAAAAGaAAAGTTGGGGAA
AGATATTTCCAATAATTTGAAGGCACTTCGcAAATTGAAAGATCACTGTCAACTTTTAAAGCACAGTCTGTCCCACTCTG
ATGTTGCTACTCTTGAAGTGGAAGGGTTAATTGATGGTGAGGACTTTGAATCTTCACTCTCTCGTGAGAAATTTGCAGAA
ATTAATCAGTCATTGTTTGACCGTTGCATGAAGCCGATAGGTACAGCCCTAAGTGATGCGGGAATTGGGAAAGAAAATAT
ACATGAGGTTATCTTGATAGGCGGATCTACAAGAATACCAAAGGTCCAGCAGCtTTTGGGGGAATACtTTTCAGAAGTTG
CCATCAGCAAGCGCATTAATCCGGACgAAGCCaTTGCAATGGGTgCTGCCATACAAGGTGCC
>HSP90
CAGTGTCAAATCGCCTCCAGCAATCACCCTGCTGTATAGTTACTAGCCAGTATGGGTGGACTGCTAATATGGAACGCATT
ATGAAGGCACAAGCATTGCGTGATTCTTCAACAATGGGCTACATGTCgGCAAAGAAGCAGCTAGAAATCAACCCGGACCA
TCCAATCATGGAAAACCTGAAGcAAAAGGTTGAGGCTGACAAGAATGACAAAACTGTCAAAAATTTAGTTTTCCTCCTCT
ATGAAACTGCTCTCTTGGCATCCGGTTTCATGTTGGAGGATCCCCAGGTGCATGCCAACCGCATTCATCGCTTGGTTAAT
TTGGGCTTGGGAATTGATGAGAGTGAGACAGTTGAAGATGACGTGCCTCAAGATATGCCATCACTGGAAGAAGGTGACGA
TGAAGCTTCAAGAATGGAAGATGTTGACTAACAACATAATTATTGGTTATGTAACTTCTAATATGATATAGCTCTAATCA
ACTTGTGCAAATATTTAGATTTAATTGTAAGCAAAAAAGGTCTAAATGATTACAGAATTCAGTTGCTATTTATaaaA
>FGF
gATCCCTGAATCAGCCATTTTCGACTCGCTTGTaAtTTTAtCTCCaTCCCTGTTTAtAGAGATCGGAAGAATGTCGCTTG
GAAAGCTATTTGGTGATATTGGAAAGTCTGTTGAGAAAGCTGCTAAAGATGTCGAGAAGGACATCGACAAATCAATCCAC
ACAATCCCTCAAAATGCATTGCAGAATGGAAATATTGTGAAACTTAGGTCAAAATCTAACAATAAGTTCCTTCGTATCTT
AGAGAATGGAACAATTGACTGTTCAGGAGACGGAGGATCAAGCTgTgAGTaCTTGGTGATtgCTTCGATGGAGCACCCTG
GCAGTCTCAAGTTCCGCAACATGGCAAACACCTTGTGGTACCTTGGTCTtGTTCAgGGGggCCCAACCaTGCCaaaCCAA
CCGCCTCCCATCTACCTAGCCGCTTCTGGTGGAGGACCAGAATGTGACTTCCTTCCTTCGATGCAGTTGGATAACTTTGT
AGCCCTCCAGTCCTCCCAGTTCCCTGGATCCCACATCGGTGCCCTTCCCAGCGGCCAGGTTACCTCCCCAACCATGACCC
CTGCCACCAGTGATGCTGCCTTCTTTAAGGTTGTGTTTCTaAGAAAACAATTTTAAACTGCAAGaAAATGCTAATTTCTG
CGTGTTCTGATCACAGTGGTGAAATACTGTAGCTCCTATGCATAATATATAAACTTTATACATATTTGTTGGCTAAGATA
TTTTGATAGTAATGTAGACTGCATTCAGTTAATGTTCATATTGtGAAAAAAtTAAgTTTgaTatC
>nNOS
GCAGCGGATTCAGAtTTGCAGAATGGAgATgAGGGTAtTCCCCtaGTCAGCAGaCAcCGTTCAGCAAGTGACTCATGTGA
tCaTGGTCAGATCATTcATGAGATGTTGAAGGCCTcTaGTgAGTTTgGGTCCATGTACCTCTTCTTTGGCTGCCGACAGA
GTACACTGGACCAcGTCTAcAAGGATGAGATGGCGAAAGCTTGCATGTTTGGAGcAGTGGAGAAATGTTATGTTGCACTT
TCAAGAGAAcCCAACCAGTCAAAgATGTATGTTCAAGATAAGCTGAAGGAGGAAGCTGATACTGTTGTCCAGTTaCTCAT
TGCTAGAGGAGcTCACTTCTATGTCTGTgGGGACATCTCaATGGCCAATGATGTtTCCCACACTCTCCATAGCATTCTGA
CTCACAACGCTGCCATGACCGACCAAGAGGCCAGAAGCTTTGTGACTGGAATGAAGGAAAATGGCACGTACCATGAAGAC
ATTTTTGGTGTTACGCTGAGGACGGCAGAAGTTACTGATAGATTTAGGACAGCTGCTAGAAGAAATTGGCAAGTCCAACT
TAGTAAGACAAGTGTCACAATAGGAGAAGAGTGTTCAGGTGAAGGAGCACAGACCAGTAGTGGACCAGCAGTGACGCTGC
TCGCTAGACATGaCGTCCCAACACAAATTAGTTACAGTTTAGAGGACAAGACACTCCCTTCTGGAAGAAAGATCTCTCAG
CATTCACACAGTGGACACCTTGTGTATTGAGATGACtTTGAGAGACACCTTCCAAGTTATAGTTGCCATAGAAATAACAT
GCTTTAAATGGTTTAGCATATAtTTTCTGATTTCGTGTACACTTTAAATCCATAGTCATTTTTACGTGCATGCTTATTGC
ATGTACATaATaaa
>Wnt
AAGTTAATAGAATCTAATGCAAACTTCTTTGCTTTggaTGTAACTATTgCTCATCAGTTgagATCAGAGAGGGGTTGGAG
GAAACTAGTGAACGGAACTGTCATCAGATACCTTCACTGCGCACTGCAGCATTAtGGTCACTATCACTGTGACATGCTGC
CGTTGTTTGAACTTGGCAGTTTACATCACCAAAGGTAtTTGGTAAACATTAAGCTGCGTTTTAtTCCTCTGAATGACTTG
ATGGAAGAGTTCAAGGGACTGTCACAATTGTCTGATGTCTCTCTCATGGAgATACATCAGACCAAAGGCTTCACTCAGGT
CTGGTGGACACTCAAGTCCGGGCTCTTTCCCATCACTGGACTCCTCCTAGCTGTGTTTATTTGGAAACTAGGTTCACGAC
AGAGGACACTTCTAGAGGGAGCTCTTATCTTTTTGGGAGCTGCAGTCACTGGTTTGGGAGTTCCTGTGGAAGTGTTATCT
CTGTACTATGACTGCCCTTGGTTAATAGTCTTCAATGATGTCCGAGTTGGTCTTGTAGTGCTAGCCCTCTCTGCCTACTG
GGTCATATTCATTGGAGAGCATTCTAAGGTGTCACCCTTGGACCATACACTGGGGAAATATTGGAAAGAACTGGCTGCAG
CCTCCCTTGGGTGCATTGTACTATTTGTGTATGAATTTTGTGAGCGAGGCATTCAAGTcGTGTATCCCTTCTTCAGTTTa
TGGTCAACTCCACACACTCAACTAATGGCAAATGTGTTTCTAGCAATATCCATCACTGCAGCAGCATTGTACAGTGTGAT

GCTGTTAGTGAGATTGGTGTCAGTGTGGTTGAGTGTTCCTGAGAAGGAGAAAGTGCTGGAAGGTATGGCAGAGATGCCCA
AAGCAGCTTATAGAAGGTCAATGTCGCAATTCAAGTTCCTCTTGACCATGTTGACAATAACAGCTGCAGTCACTTTATCA
ACTTTCATACCAACTGAGATACATGACTTTGGAATGGAGGCTTCAGCCGCAACCATGTCCGTATACAGTGGTATACTGAC
AGGGAGTTAtGCCCTTTGGAATGTGTATACTGTGATAGTCtTAGTGGAGCACATAATCGGGGTTCAAGATCTGTTTCGTT
TAACTTaATGTGCTAGTCGCTCACAGTaGCTACTTTGTGTGGTTGTTAGTGGAGACCAATTGCTACtATGTTGTGCACAT
GTGCTCTTGTGATGCAGATAAGTCCAGATTGATTGGATATACAGCAGAAAGACTTTTAAACAAAGGCTCGCAAGAATAGA
CACTTATGCTACATTGTCACTTACTTCAGTATCAAGTATACATGATTATGTTAATATGAATACACTGAGTAGAGTGATGT
GCTTTGAAGTGTTCtATTCtCCTg

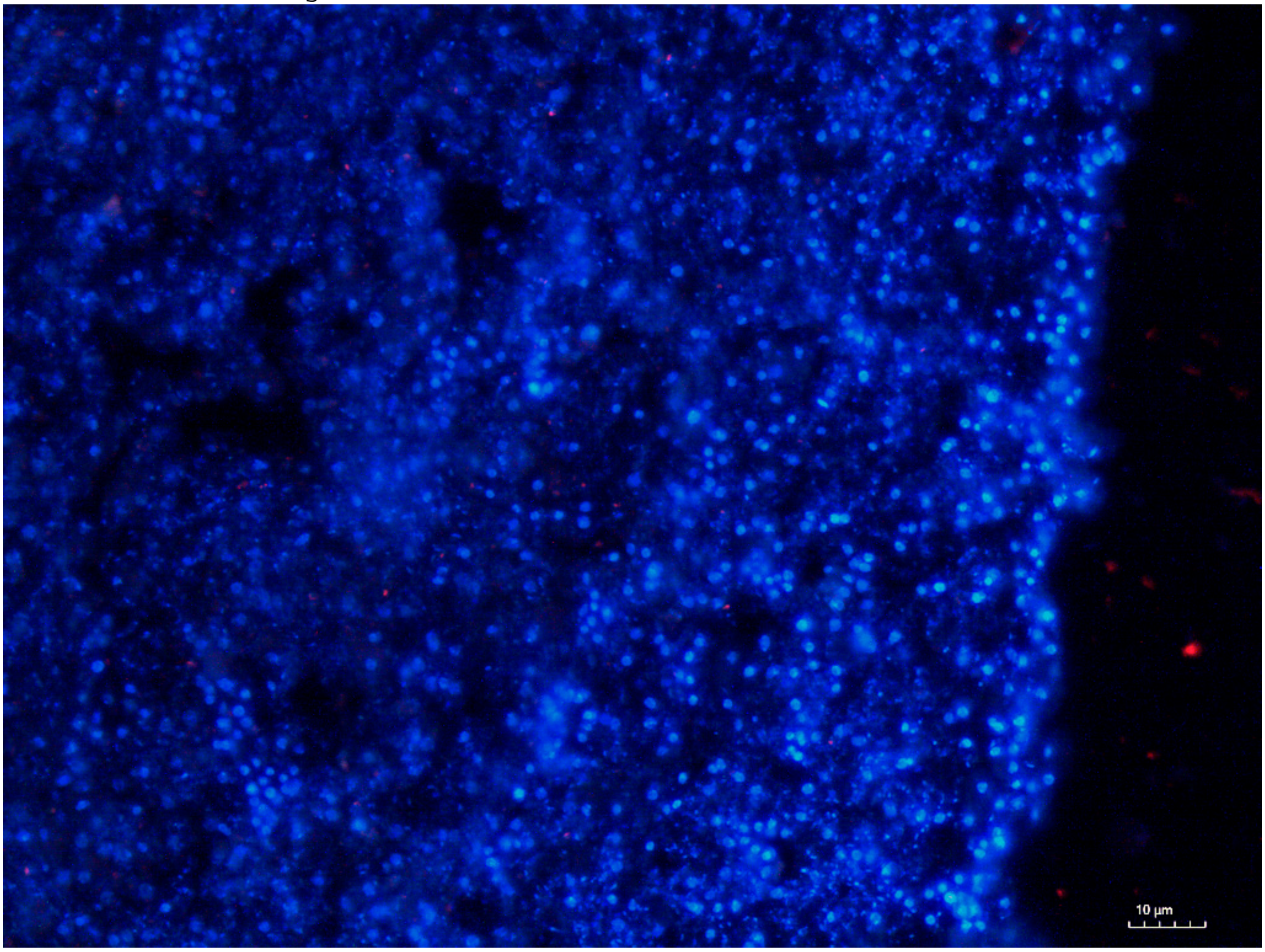

Figure S1. Negative control of Figure 3. Histological sections showing the regenerating edges of *Chondrosia reniformis* fragments 24 h after being cut.

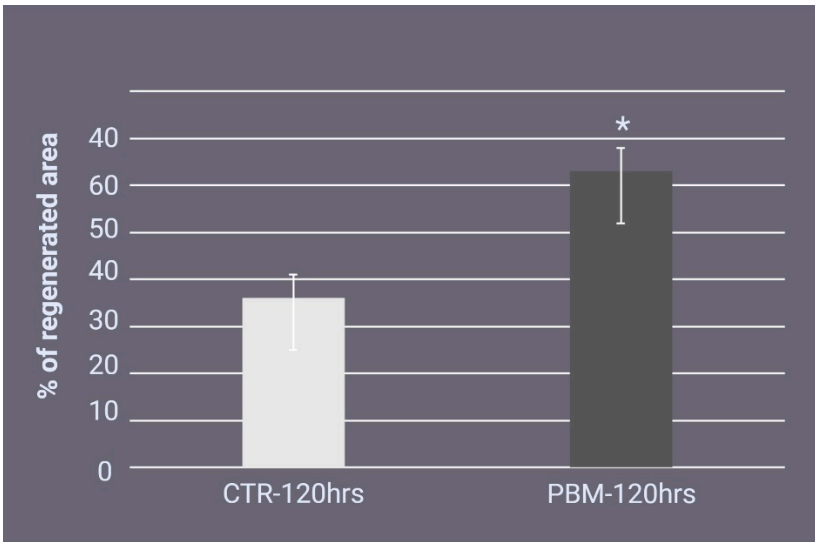

Figure S2. Percentage of regeneration at 120 hours (hrs) from cutting. The asterisks indicate significant differences between groups based on the t-student test, *$p < 0.05$. The program ImageJ was employed to investigate surface areas [69].
